# Efficacy and Safety of Oral Semaglutide in the Management of Diabetes and Obesity: A Comprehensive Meta-analysis of Real-world Evidence

**DOI:** 10.17925/EE.2026.22.1.8

**Published:** 2026-05-14

**Authors:** Sweekruti Jena, Radhika Jindal, Deep Dutta, Abul Bashar Mohammad Kamrul-Hasan, Kunal Mahajan

**Affiliations:** 1. Department of Endocrinology, Kalinga Hospital, Bhubaneshwar, Odisha, India; 2. Department of Endocrinology, Vardhman Mahavir Medical College & Safdarjung Hospital, New Delhi, India; 3. Department of Endocrinology, Center for Endocrinology Diabetes Arthritis & Rheumatism (CEDAR) Superspeciality Healthcare, Dwarka, New Delhi, India; 4. Department of Endocrinology, Mymensingh Medical College, Mymensingh, Bangladesh; 5. Himachal Heart Institute, Mandi, Himachal Pradesh, India

**Keywords:** Glucagon-like peptide 1 receptor agonists, hypoglycemic agents, obesity, observational study, oral drug administration, semaglutide, treatment outcome, type 2 diabetes, weight loss

## Abstract

**Background:**

Oral semaglutide is the only oral glucagon-like peptide-1 receptor agonist approved for type 2 diabetes (T2D) management. Although its efficacy and safety are established from randomized controlled trials (RCTs), real-world evidence (RWE) may differ. No comprehensive systematic review and meta-analysis (SRM) has holistically analysed the RWE on oral semaglutide. This SRM analysed the RWE outcomes of oral semaglutide.

**Methods:**

Electronic databases were searched for real-world studies reporting outcomes in adults with T2D receiving oral semaglutide. Primary outcomes were changes in glycated haemoglobin (HbA1c) at 6 and 12 months. Secondary outcomes included changes in body mass index, blood pressure, lipids and adverse events (AEs). Subgroup analyses compared outcomes between Asian and non-Asian cohorts.

**Results:**

A total of 59 studies (24,859 individuals) were analysed. The pooled mean HbA1c reduction was -1.11% (95% confidence interval: -1.37 to -0.86; I²=98.8%) at 6 months and -1.19% at 12 months, with 54.7% achieving HbA1c <7%. Weight reduction averaged -4.38 kg at 6 months and -5.96 kg at 12 months. Significant improvements were observed in cardiometabolic parameters. The pooled prevalence of total AEs, gastrointestinal AEs, nausea, diarrhoea and hypoglycaemia was 28.9%, 20.5%, 12.5%, 5.9% and 2.2%, respectively. Discontinuation and dose reduction due to AEs occurred in 8.7% and 4.2% of patients, respectively. Efficacy and safety outcomes were consistent across regions (p for subgroup >0.05).

**Conclusion:**

Oral semaglutide demonstrates robust real-world efficacy and safety in T2D and obesity management, similar to RCTs, consistent across Asian and non-Asian populations.

Glucagon-like peptide-1 receptor agonists (GLP-1RAs) and GLP-1RA-based therapies are currently considered to be one of the most attractive classes of medications for managing type 2 diabetes (T2D) because of their impressive weight loss properties, glycated haemoglobin (HbA1c) reduction, beneficial impact on blood pressure, lipids, metabolic dysfunction-associated steatotic liver disease, renal outcomes, cardiovascular protection and reduction in heart failure.^1–3^ These cardiorenal and metabolically beneficial properties of GLP-1RAs make them ideal for managing the global pandemic of diabesity.^4^

Among all the GLP-1RAs available for clinical use, only semaglutide is available as an oral preparation. Oral semaglutide was approved for clinical use by the US Food and Drug Administration (FDA) and the European Medicines Agency (EMA) in September 2019 and April 2020, respectively.^5 6,7^ Oral semaglutide helps to overcome the injection barrier of subcutaneous GLP-1RAs across many ethnic groups worldwide, and thus enhances convenience and patient acceptance.^8^ Oral semaglutide has a unique administration profile, requiring it to be taken on an empty stomach and to wait a 30 minute post-dose interval before eating or taking other medications to ensure adequate and maximal drug absorption and bioavailability for clinical efficacy; issues that are not seen with once-weekly injectable semaglutide.^8^ Hence, oral and injectable semaglutide are practically different medicines, and the clinical efficacy/safety data of one preparation cannot be extrapolated to the other. Since there are no meta-analysis on real world use of oral semaglutide, we aimed to quantitatively analyse the efficacy and safety of oral semaglutide use in RWSs.

## Methods

### Ethical compliance

This article adhered to the standardized methodology described in the Cochrane Handbook for Systematic Reviews and was reported following the Preferred Reporting Items for Systematic Reviews and Meta-Analyses (PRISMA) guidelines.^9^ The study was registered with PROSPERO (International Prospective Register of Systematic Reviews; registration number: CRD420251154717), with open online access to the protocol summary.^10^

### Search strategy

A comprehensive database search was conducted in PubMed, Ovid Embase, Ovid Medline, Cochrane Library, ClinicalTrials. gov, China National Knowledge Infrastructure (CKNI), ctri.nic.in and Google Scholar up to September 2025. We used key terms such as ‘oral semaglutide’ and ‘diabetes’ OR ‘obesity’ combined with Boolean operators to refine retrieval. References in the published articles were also screened manually to identify eligible studies.

### Eligibility criteria

The PICOS (Population, Intervention, Comparator, Outcomes, and Study design) criteria were utilized to screen and select the studies. The population (P) included people living with T2D and/or with obesity. The intervention (I) consisted of the use of oral semaglutide along with the standard of care for managing T2D/obesity. Oral semaglutide was administered once daily at doses of 3, 7 and 14 mg. Recently investigated higher dose oral semaglutide for obesity management, as well as newer oral formulations with alternative dose strengths (e.g. 1.5, 4 and 9 mg once daily), were not included in this meta-analysis due to the lack of corresponding real-world evidence at the time of analysis.^11,12^ The control (C) group (if available) used placebo or any other approved medication for T2D/obesity.^13–16^ In addition, studies without a control group were considered for single-arm and proportion meta-analysis. The outcomes (O) focused on HbA1c reduction, weight reduction, per cent weight reduction, ability to achieve HbA1c <7%, hypoglycaemia and adverse effects. The study type (S) comprised RWSs (both single-armed and double-armed studies). Cross-sectional studies, case reports, reviews, expert opinions, editorials, letters to the editor and duplicate reports were excluded from the analysis. Duplicates were removed before screening articles by title and abstract, followed by full-text screening to confirm eligibility.

### Study outcomes

#### Primary outcome

The primary outcome was the mean change in percentage HbA1c from baseline to 6 and 12 months follow-up after starting oral semaglutide.

#### Secondary outcomes

Secondary outcomes were pre-specified to evaluate the metabolic and safety effects of oral semaglutide. These included:

- Glycaemic efficacy: proportion of participants achieving HbA1c <7%.
- Weight outcomes: mean change in body weight (kg) and body mass index (BMI; kg/m^2^); and proportion of patients achieving ≥3% and ≥5% weight reduction, respectively.
- Cardiometabolic outcomes: mean change in systolic blood pressure (SBP; mmHg) and diastolic blood pressure (DBP;, mmHg) and total cholesterol (mg/dL).
- Safety and tolerability outcomes: pooled prevalence of total adverse events (TAEs), serious adverse events (SAEs), discontinuation or dose reduction due to adverse events (AEs), gastrointestinal AEs (nausea, diarrhoea) and hypoglycaemia (any/severe).

### Study selection

Two reviewers independently evaluated all identified articles by first screening titles and abstracts, followed by full-text review. Studies were included if they met the pre-defined criteria, and any uncertainty was resolved through discussion with a third author.

### Data extraction

Data extraction was carried out independently by two authors using a structured extraction sheet for this article. When multiple publications originated from the same study population, data were merged and analysed as a single set. The variables included first author, publication year, the country of origin, study design, methodology, anthropometric data, diabetes duration, HbA1c, medication profile and the pre-specified outcomes. Inconsistencies were settled by consensus.

### Data synthesis and statistical analysis

Statistical analysis was performed using RStudio (version 2025.4.5.0; Posit Software, Public Benefit Corporation, Boston, MA, USA) with the meta and metafor packages in R (R Foundation for Statistical Computing, Vienna, Austria).^17^ Continuous outcomeswere summarized using mean values and standard deviations, while categorical outcomes were expressed as frequencies or proportions. Effect sizes for continuous parameters were reported as mean differences, and categorical outcomes were analysed using odds ratios or risk ratios, each presented with 95% confidence intervals (CIs). In the studies with an active control group, changes from baseline values of the continuous and categorical outcomes in the semaglutide versus control groups for the categorical variables were calculated. For the single-arm cohorts, we calculated the mean change from baseline for all available outcomes and pooled these estimates across included studies. Because the included studies varied in design, population characteristics and follow-up duration, a random-effects model was selected as the primary analytical framework. The analysis applied a restricted maximum-likelihood approach to estimate τ², while CIs for τ and τ² were derived using the Q-profile method. Heterogeneity (I²) was calculated from the Q statistic using raw, untransformed means. Pre-specified subgroup analysis was performed to explore regional differences in treatment response and safety. Each study was categorized by its geographical origin as either Asian or RoW (non-Asian) based on its country of origin. Subgroup meta-analyses were conducted wherever adequate data were available. All analyses were performed in R using the meta package. For consistency across the forest plots, author names appear as they were exported from the original datasets (e.g. Jimenez B = Rodríguez Jiménez B; Roychoudhury S = Roy Chowdhury S; Roychowdhury S = RoyChaudhuri S; Vidas M = Marques Vidas M; Houtum = van Houtum). Full and correct author names are used in the manuscript text and reference list.

### Assessment of heterogeneity and publication bias

Heterogeneity was quantified using I² statistics, with values below 30% reflecting low variability, 30–75% suggesting moderate variation and >75% indicating substantial heterogeneity.^18^ Publication bias was assessed visually using funnel plots and statistically using Egger’s regression; where applicable, assessment of heterogeneity was initially performed by examining forest plots.

### Assessment of the quality of the included studies

Study quality was examined using the Risk Of Bias In Non-randomized Studies of Interventions, Version 2 (ROBINS-IV2) for both RCTs and non-randomized intervention trials.^19^ The ROBINS-IV2 tool evaluates seven domains, including confounding, participant selection, intervention classification, deviations, missing outcome data, measurement bias and reporting accuracy. Each study received an overall rating of low, moderate or high risk based on domain-level judgement. In case of discrepancies, the third and fourth authors reached a consensus. Risk-of-bias plots were created using the Risk-of-bias VISualization (robvis) web application (version 0.3.0; University of Bristol, Bristol, UK).^20,21^

## Results

**Figure 1:**
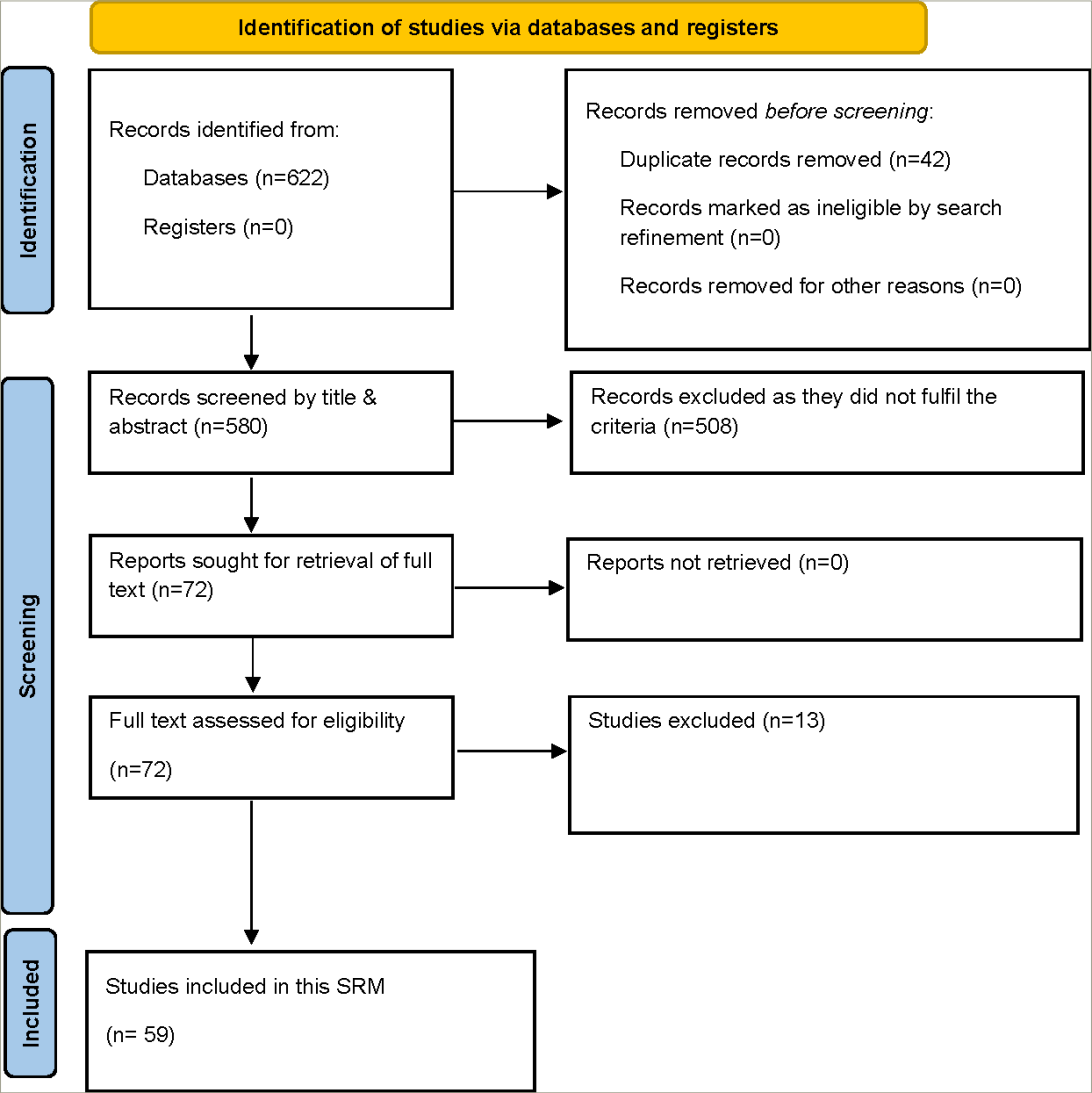
Flowchart elaborating on study retrieval and inclusion in this systematic review and meta-analysis

An initial search revealed 622 articles. Forty-two duplicates were removed. Following screening of the titles and the abstracts, the search identified 72 eligible articles, which were evaluated in detail for consideration for inclusion in this systematic review and meta-analysis (SRM). Finally, data from 59 articles, which fulfilled all inclusion and exclusion criteria, were analysed in this SRM.^22–78^ The flow of patients has been elaborated in the PRISMA flow chart (*Figure 1*).

### Study characteristics

The characteristics of the different studies analysed in this SRM have been elaborated in *Table 1*.^22–80^ *Table 2* shows the different medications received by the patients in different studies.^22–27,29–32,34–37,40–43,45–50,52,56–59,62,64,65,67–69,71,74,75,77,78^ maximum number of analysed articles was from Italy (n=16), followed by Japan (n=9), India (n=8), the USA (n=6), Spain (n=5), two articles each from the UK, Switzerland and the Middle East (United Arab Emirates, Saudi Arabia and Kuwait) and one article each from Canada, Croatia, China, Denmark, Sweden, the Netherlands, Poland, Romania and Slovenia (*Table 1*). Subgroup analysis of data from the articles was performed comparing the outcomes in Asians (n=20) with Europeans (n=32) and North Americans (n=7).

### Risk of bias and quality assessment of the included studies

The risk of bias was calculated to be low among the studies analysed in this SRM. Details have been elaborated on in *Supplementary* Figure S1.

### Study characteristics and baseline profile

Outcomes have been presented for 59 studies involving 28,479 individuals with T2D on oral semaglutide therapy.^22–80^ The pooled mean age was 61.0 years (95% CI: 59.24–62.75; I²=99.6%; SF-S2). The pooled mean baseline weight was 89.96 kg (95% CI: 86.65–93.27; I²=99.6%; *k*=47 studies; SF-S3), and baseline BMI was 31.77 kg/m² (95% CI :30.86–32.68; I²=99.5%; SF-S4). Baseline HbA1c was 8.08% (95% CI: 7.90–8.27; I²=98.9%; SF-S5). The mean duration of T2D was 9.92 years (95% CI: 8.93–10.91; I²=98.7%; SF-S6).

### Glycaemic outcomes

**Figure 2:**
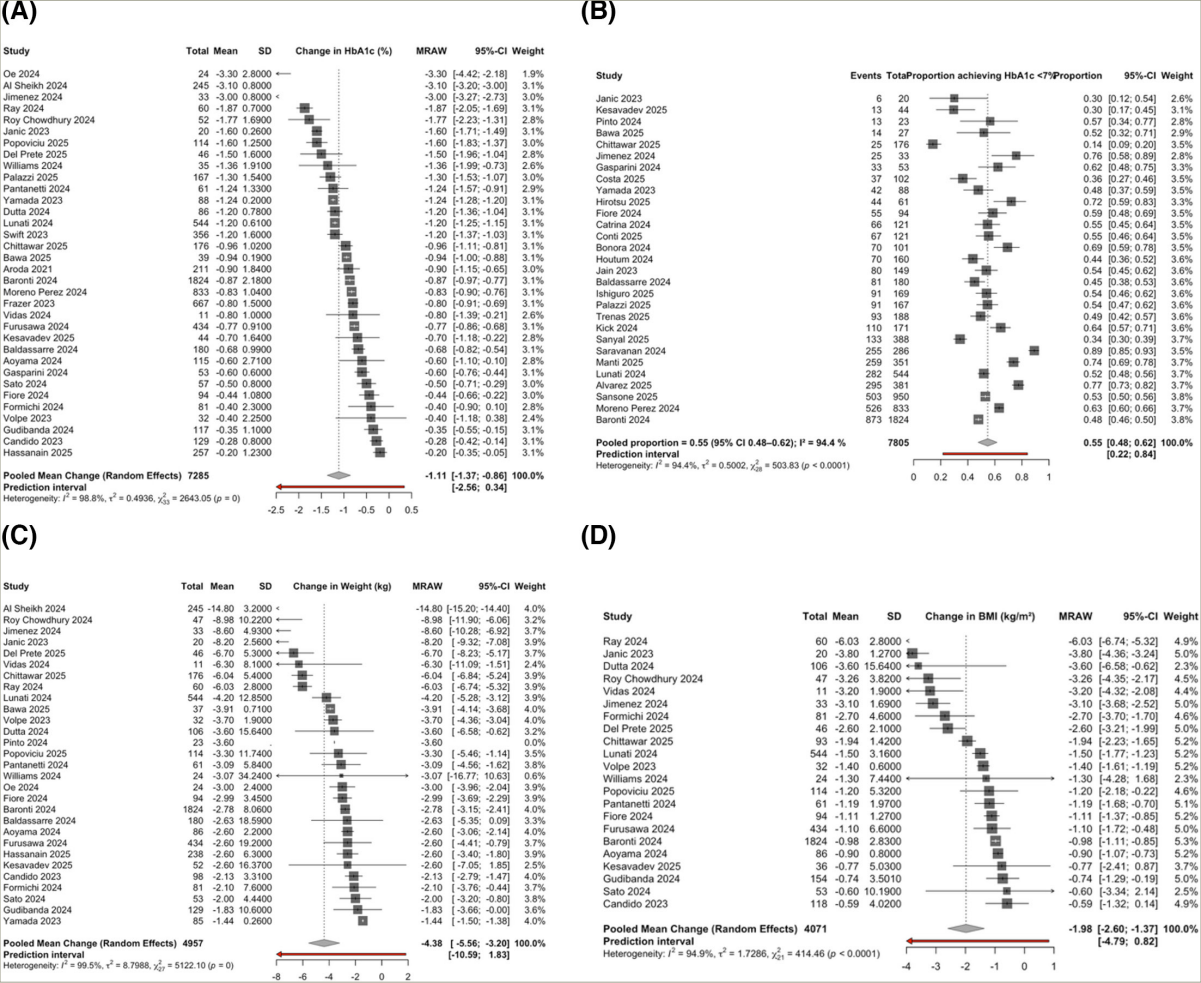
Glycemic and weight outcomes with oral semaglutide^22–80^

After 6 months’ use of oral semaglutide, pooled HbA1c reduction was -1.11% (95% CI: -1.37 to -0.86; I²=98.8%; *k*=34; *Figure 2A*).^22–80^ At 12 months, the pooled HbA1c reduction was -1.19% (95% CI: -1.46 to -0.92; I²=99.2%; *k*=24). The pooled chances of achieving HbA1c <7% were 54.7% (95% CI: 47.6–61.6%; I²=94.4%; *k*=29; *Figure 2B*) following oral semaglutide use.

*SRM = systematic review and meta-analysis*

**Table 1:** Baseline characteristics of studies included for the meta-analysis^22–80^

Study details	Major inclusion criteria	Primary outcomes	N	Female (%)	Age (years), mean ± SD or median (IQR)	Weight (kg), mean ± SD or median (IQR)	BMI (kg/m^2^), mean ± SD or median (IQR)	HbAlc (%), mean ± SD or median (IQR)	Duration of diabetes (years), mean ± SD or median (IQR)	Study duration
Alsheikh et al., 2024;^23^ retrospective observational; Saudi Arabia	Adults with T2D	Change in HbA1c	245	49.8	54.6 ± 8.4	113.1 ± 20.6	38.2 ±6.9	10.1 ±1.2	12.2 ±4.6	12 months
Delgado Alvarez et al., 2025;^24^ phase IV multicentric prospective observational; Spain	Adults with T2D	Change in HbA1c	458	40	59.4 ± 11.4	100.6 ±20	36.4 ±6.4	7.8 ± 1.6	6.6 ±7.2	34-44 weeks
Aoyama et al., 2024;^25^ retrospective observational; Japan	Adults with T2D	Changes in cardiometabolic parameters	153	30	61 ± 7.1	77.9 ±6.5	28.2 ± 5	7.9 ± 4.07	NR	6 months
Aroda et al., 2021;^26^ retrospective observational; USA	Adults with T2D	Change in HbA1c	782	54.5	57.8 ± 11.3	104.9 ±24	36.2 ±7.6	8.4 ± 1.8	6.9 ±4.8	6 months
Baldassarre et al., 2024;^27^ retrospective observational; Italy	Adults with T2D	Change in HbA1c and body weight	192	44	67 (15)	82.47 ± 17.63	30.41 ± 5.86	7.9 ± 1.19	9 (12)	6 months
Baronti et al., 2024;^28^ retrospective observational; Italy	Adults with T2D		1,824	40.8	65.4 ± 10.9	88.6 ± 19.2	31.2 ±5.9	8 ± 1.6	11.1 ± 8.2	12 months
Bawa et al., 2025;^29^ retrospective observational; India	Adults with T2D	Change in HbA1c	51	35.3	51.22 ± 11.78	97.4 ± 17.5	NR	8.16 ± 1.37	10.79 ± 6.83	12 months
Bonora et al., 2024;^30^ retrospective observational; Italy	Adults with T2D	Change in HbA1c	166	35.5	64.4 ± 8.6	81.7 ± 16.9	28.9 ±5.3	7.5 ± 1.3	10.1 ± 8.2	18 months
Candido et al., 2023;^31^ retrospective observational; Italy	Adults with T2D	Change in HbA1c	129	43	72 (66-79)	NR	28.8 (26.3-32.8)	7.2 (6.6-8)	11 (6-22)	6 months
Catrina et al., 2024;^32^ phase IV multicentric prospective observational; Sweden	Adults with T2D	Change in HbA1c	187	35.3	62.5 ± 10.98	97.3 ± 19.5	32.4 ±5.8	7.7 ± 1.2	6.8 ±5.7	34-44 weeks
Chittawar et al., 2025;^33^ retrospective observational; India	Adults with T2D	Change in HbA1c	340	40.3	48.11 ±11.44	94.49 ± 19.09	33.56 ± 6.08	8.59 ± 1.27	-	30 weeks
Conti et al., 2025;^80^ retrospective observational; Italy	Adults with T2D	Medication persistence	121	42	67 ± 11	NR	30.5 ±5.6	7.7 ± 1.1	10.8 ±8.7	18 months
Costa et al., 2025;^34^ retrospective observational; Italy	Adults with T2D	Change in HbA1c	104	41.4	68.9 ± 9.9	83.2 ± 15.7	30.5 ±5.2	7.62 ± 0.96	15.7 ±8.7	37 weeks
Del Prete et al., 2025;^35^ retrospective observational; Italy	Adults with obesity or overweight and T2D	Change in weight, BMI, HbA1c	46	52.1	65.7 ± 12.8	95.2 ± 15	34.3 ±5.3	7.6 ± 1.6	NR	24 months
Dutta et al., 2024;^36^ retrospective observational; India	Adults with T2D and prediabetes	Effectiveness (glycaemic metrics, weight, body composition), safety and tolerability	351	49	53(43-61)	91 (79.2-103)	32.74 (29.34-36.64)	7.9 (6.9-9)	10 (5-16)	Four follow-up visits (up to 351 days)
Fadini et al., 2025;^37^ retrospective observational; Italy	Adults with T2D	Change in HbA1c	105	35.2	64.6 ± 8.7	82.9 ±17.9	29.1 ± 5.5	7.6 ± 1.1	10.1 ± 7.9	18 months
Fiore et al., 2024;^79^ retrospective observational; Italy	Adults with T2D, age >65 years	Change in HbA1c	101	63.4	74.7 ± 6.1	76.8 ± 14.1	28.7 ±4.7	7.4 ± 0.88	8± 5.1	6 months
Formichi et al., 2024;^38^ retrospective observational; Italy	T2D, oral versus subcutaneous semaglutide	Not mentioned	115	42	65.2 ± 10.4	80 ± 15.2	29.1 ± 4.7	7.7 ± 1.3	12.8 ± 10.2	6 months
Frazer et al., 2023;^39^ retrospective observational; USA	Adults with T2D	Change in HbA1c	1,012	50	59 ± 11.8	NR	NR	8.2 ± 1.7	NR	6 months
Furusawa et al., 2024;^40^ retrospective observational; Japan	T2D, age >20 years	Changes in HbA1c, safety assessed byanyAEs leading to drug discontinuation	434	44	55.5 ± 12.6	80.2 ± 19.2	29.6 ± 6	7.65 ± 1.11	NR	12 months
Gasparini et al., 2024;^41^ retrospective observational; Croatia	Adults with T2D	Change in cardiovascular risk factors	53	50.94	65 ±9	NR	31.4(29.7-34.9)	7.6 (7.0-8.2)	5(2-11)	9 months
Gudibanda et al., 2024;^43^ retrospective observational; India	Adults with T2D	Change in HbA1c, BMI and body weight	188	48.4	51.31 ±11.07	91.1 ± 10.46	35.22 ± 3.53	8.11 ±0.84	NR	160 days
Hassanein et al., 2025;^43^ multicentric prospective observational; UAE, Saudi Arabia, Kuwait	Adults with T2D	Change in HbA1c	257	42	52.94 ± 9.7	89.8 ± 18.7	32.3 ±5.8	6.8 ± 1.2	NR	20 weeks
Hirotsu et al., 2025;^44^ prospective pilot study; Japan	Adults with T2D, HbA1C 7-10.5%, switched from DPP-4i	Baseline characteristics, and differences in haematological tests between those who achieved HbA1c <7% and those who did not	61	34	61.7 ± 3.6	NR	27.3 ± 1.01	8.02 ± 0.23	16.25 ± 2.8	NR
Horii et al., 2024;^45^ retrospective observational; Japan	Adults with T2D	Frequency of discontinuation of semaglutide over 12 months	6,140	41.3	60.5 ± 14.2	NR	29.6 ±6.3	NR	NR	12 months
van Houtum et al., 2024;^46^ phase IV multicentric prospective observational; Netherlands	Adults with T2D	Change in HbA1c	187	46	58.8 ± 11.36	103.1 ± 19.51	35.1 ± 5.9	8.6 ± 1.28	8.7 ±5.92	34-44 weeks
Ishiguro and Ishiguro, 2025;^47^ retrospective observational; Japan	T2D, HbA1C >7%	Changes in mean HbA1c at 180 days, HbA1c reduction to <7%, weight reduction >3%	169	29.6	63.0 (54.0-70.0)	75.0 (66.0-87.4)	27.2 (24.4-30.9)	7.7 (7.2-8.3)	10.0 (5.0-17.0)	180 days
Jain et al., 2023;^48^ multicentric prospective observational; Canada	Adults with T2D	Change in HbA1c	182	35.7	58.6 ± 10.9	93.7 ± 22.7	32.5 ±6.7	8 ± 1.36	8.1 ± 6.9	34-44 weeks
Janic et al., 2023;^49^ prospective open-label interventional; Slovenia	Adults with T2D, age 30-65 years, HbA1C>7%, BMI >30 kg/m^3^	Change in glycaemic parameters and body weight	20	45	59.9 ± 1.5	100.9 ±2.7	34.6 ± 1.4	9.4 ± 0.3	8.5 ± 1.4	3-5 months
Rodríguez Jiménez et al., 2024;^50^ retrospective observational; Spain	Adults with T2D and obesity (BMI >30 kg/m^3^)	Change in body fat mass	33	33.33	61.8 ±7	94.8 ± 15.6	33.2 ±3.9	9.3 ± 1.9	9.6 ±6.3	24 weeks
Kesavadev et al., 2025;^51^ retrospective observational; India	Adults with T2D	Change in HbA1c and BMI	52	32.7	50.79 ± 14.95	81.79 ± 17.56	30.05 (25.59-32.89)	8 (7.1-9.5)	NR	6 months
Kick et al., 2024;^53^ multicentric prospective observational; Switzerland	Adults with T2D	Change in HbA1c	185	36.2	62 ± 10.4	95.6 ± 17.9	33.2 ±4.8	7.7 ± 1.5	6.4 ±5.3	34-44 weeks
Krajnc et al., 2025;^53^ retrospective observational; Poland	Adults with obesity, without diabetes	NR	93	57	51.3 ± 12.5	105 ± 18	35 ±6	NR	NR	1 year
Kwon et al., 2025;^54^ retrospective observational; USA	Adults with T2D	Change in weight	57	31.6	60.7 ± 10.5	90.67	NR	NR	NR	2 years
Lunati et al., 2024;^55^ retrospective observational; Italy	Adults with T2D	Change in HbA1c	544	NR	NR	80.4 ± 13.5	28.6 ±3.3	7.6 ± 0.7	NR	6 months
Lv et al., 2024;^56^ retrospective; USA	Adults with T2D	Adherence and persistence to index medication over 12 months	5,485	48.1	52.7 ± 9.3	NR	NR	NR	NR	12 months
Manti et al., 2025;^57^ phase IV multicentric prospective observational; Italy	Adults with T2D	Change in HbA1c	445	40	62.9 ± 10.2	NR	31.2 ±6	7.8 ± 1.3	8 ± 6.9	34-44 weeks
Moreno-Perez et al., 2024;^58^ retrospective observational; Spain	Adults with T2D	Weight reduction >5%, HbA1c reduction >1%	1,018	46.1	63 (56-70)	94 (83.7-108)	33.8(31.1-38.7)	7.8 (6.9-8.8)	7.5 (6.9-8.8)	12 months
Oe et al., 2024;^59^ retrospective observational; Japan	T2D, age >65 years, HbA1c >7%	Change in HbA1c	30	50	76.2 ± 7.3	56.4 ± 10.8	23.4 ±3.1	8 ± 2.9	17.0 (9.5-24.0)	6 months
Palazzi et al., 2025;^60^ observational; Italy	T2D	Reduction in HbA1c and body weight	167	46	69 (12)	83.3 (19)	29.6 (7.1)	8.4 ± 1.5	7.6 ±4.2	3 months
Pantanetti et al., 2024;^61^ prospective observational; Italy	Adults with T2D	Change in HbA1c, PPG, body composition, body weight, BMI	61	21.4	61 ± 9.9	89.19 ±5.84	30.81 ± 1.96	7.92 ± 1.33	4.67 ± 3.93	6 months
Pinto et al., 2024;^62^ retrospective observational; USA	Adults with T2D	Change in HbA1c and weight	23	47.8	59 (55-64)	110 (82.8-124.4)	35.7(28.5-41.5)	8.2 (7-10.2)	NR	6 months
Popoviciu et al., 2025;^63^ prospective observational; Romania	Adults with T2D (HbA1C>7.2%) and obesity (BMI >25 kg/nr)	Change in HbA1c	114	40.4	59.37 ± 9.28	95.56 ± 11.75	34.95 ± 5.35	8.83 ± 1.44	6.51 ± 2.74	6 months
Ray et al., 2024;^64^ retrospective observational; India	Adults with T2D	Change in HbA1c	80	30	46.6 ± 8.1	82.27 ± 22.2	NR	8.67 ±1.3	5.8 ±3.9	18 months
Roy Chowdhury et al., 2024;^65^ retrospective observational; UK	Adults with T2D	Change in HbA1c	53	51	58.45 ± 10.35	109.74 ± 27.74	39.28 ± 10.17	9.28 ± 1.29	NR	6 months
RoyChaudhuri et al., 2023;^66^ retrospective observational; India	Adults with T2D	Change in glycaemic metrics on AGP	10	60	58.3 ± 11.26	NR	30.3 ± 4.03	NR	NR	4 weeks
Sansone et al., 2025;~ retrospective observational; Italy	Adults with T2D	Change in HbA1c	950	63.7	68.3 ± 10.1	82.5 ± 17.6	NR	8 ± 1.3	13.9 ±9	12 months
Sanyal et al., 2025;^67^ multicentric prospective observational; India	Adults with T2D	Change in HbA1c	388	44.8	50.1 ± 10.6	89 ± 16.7	33.1 ± 5.6	9 ± 1.49	6.3 ±5.8	34-44 weeks
Saravanan et al., 2024;^68^ multicentric prospective observational; Switzerland	Adults with T2D	Change in HbA1c	333	38.7	58.5 ± 11.94	102.8 ±24.13	35.5 ± 7.96	8.6 ± 1.44	7.2 ±5.9	34-44 weeks
Sato et al., 2024;^69^ retrospective observational; Japan	Adults with T2D on 14 mg oral semaglutide	Change in HbA1c	66	25.8	56.1 ± 12.1	90 ± 20.5	32 ± 7.2	7.4 ± 1	11.3 ±7.2	24 weeks
Swift et al., 2023;^70^ retrospective observational; USA	Adults with T2D	Change in HbA1c	520	41	57.8 ± 11.3	NR	NR	8.8 ± 1.5	NR	6 months
Trenas et al., 2025;^71^ prospective; Spain	HFpEF, T2D, obesity	HF health status, change in body weight at 18 months	202	58.4	77.5 ± 12	95.4 ± 16.4	34.1 ± 3.1	7.8 ± 1.2	14.1 ± 7.3	18 months
Marques Vidas et al., 2024;^72^ retrospective observational; Spain	T2D, age >18 years, CKD with eGFR >15 mL/min/1.73 nr	Change in HbA1c and weight	19	26.3	68.6 ± 7.4	96.7 ± 14.5	33.4 ±3.5	7.2 ± 1.1	12.1 ± 6.1	6 months
Volpe et al., 2023;^73^ prospective observational; Italy	Adults with T2D	Change in body composition	32	56.7	66.3 ± 8.5	75.3 ± 10.8	28.2 ±3.3	6.4 ± 2.7	8.7 ±7.6	6 months
Weinreich et al., 2025;^74^ retrospective observational; Denmark	Adults with T2D	Efficacy and safety	96	26	63.8 ± 12.7	97.4 ±21.3	NR	8.5 ± 3.7	16.45 ± 8.22	1 year
Williams et al., 2024;^75^ retrospective observational; UK	Adults with T2D	Reasons for oral semaglutide initiation, changes in body weight, BP, glycaemic control, lipid profile, safety data	76	50	59.3 (51.4-67.6)	98.2 (85.1-110.1)	34.6 (30.7-37.6)	9.37 ± 1.59	13.0 (8.5-19.0)	12 months
Xiong et al., 2024;^76^ retrospective; China	Adverse drug events of oral semaglutide, from FDAAE reporting system	Adverse drug events of oral semaglutide	2,398	56	64.0 (54.5-72.0)	86.0 (71.0-102.7)	NR	NR	NR	NR
Yabe et al., 2025;^77^ multicentric prospective observational; Japan	Adults with T2D	Change in HbA1c	624	43	64.1 ± 14.1	72.4 ± 16.1	27.5 ± 5	7.7 ± 1.1	10.7 ±8.5	34-44 weeks
Yamada et al., 2023;^78^ retrospective observational; Japan	Adults with T2D	Change in HbA1c and body weight	88	37.5	62 (53.8-68)	73.6 ± 1.58	27.3 ±0.61	8.53 ±0.17	10.5 (5-18)	6 months

*AE = adverse events; AGP = ambulatory glucose profile; BMI = body mass index; BP = blood pressure; CKD = chronic kidney disease; DPP-4is = dipeptidyl peptidase inhibitors; eGFR = estimated glomerular filtration rate; FDA = Food and Drug Administration; FPG = fasting plasma glucose; HbAtc - glycated haemoglobin; HF - heart failure; HFpEF - heart failure with preserved ejection fraction; ID = study identification; IQR = interquartile range; NR = not reported; SD = standard deviation; 72D = type 2 diabetes.*

**Table 2:** Profile of co-medications taken by patients along with semaglutide in the real-world studies^22–27,29–32,34–37,40–43,45–50,52,56–59,62,64,65,67–69,71,74,75,77,78^

Author, year	Number of patients on semaglutide	Biguanides (%)	SGLT2i (%)	Insulins (%)	SUs (%)	TZDs (%)	AGIs (%)
Alsheikh et al., 2024^23^	245	113 (46.12)	115 (46.94)	129 (52.65)	121 (49.39)	NR	NR
Delgado Álvarez et al., 2025^24^	458	224 (48.90)	114 (24.89)	NR	32 (6.98)	4 (0.87)	NR
Aoyama et al., 2024^25^	153	114 (74.51)	109 (71.24)	14 (9.15)	24 (15.69)	13 (8.59)	11 (7.19)
Aroda et al., 2021^26^	782	456 (58.31)	199 (25.44)	NR	232 (29.67)	61 (7.80)	NR
Baldassarre et al., 2024^27^	192	167 (86.98)	72 (37.5)	20 (10.42)	9 (4.69)	19 (9.89)	NR
Bawa et al., 2025^29^	51	39 (76.48)	12 (23.53)	30 (58.82)	23 (45.09)	NR	NR
Bonora et al., 2024^30^	166	154 (92.77)	35 (21.08)	10 (6.02)	12 (7.22)	7 (4.21)	NR
Candido et al., 2023^31^	129	84 (65.11)	68 (52.71)	36 (27.91)	17 (13.18)	11 (8.53)	NR
Catrina et al., 2024^32^	187	147 (78.60)	40 (21.39)	NR	12 (6.41)	9 (4.81)	NR
Costa et al., 2025^34^	104	NR	NR	NR	104 (100)	NR	NR
Del Prete et al., 2025^35^	46	39 (84.78)	NR	NR	NR	NR	NR
Dutta et al., 2024^36^	351	285 (81.19)	203 (57.83)	95 (27.06)	169 (48.15)	12 (3.42)	NR
Fadini et al., 2025^37^	105	98 (93.33)	NR	7 (6.67)	11 (10.48)	4 (3.81)	NR
Furusawa et al., 2024^40^	434	297 (68.43)	300 (69.12)	19 (4.37)	74 (17.05)	21 (4.84)	32 (7.34)
Gašparini et al., 2024^41^	53	44 (83.01)	16 (30.19)	7 (13.21)	16 (30.19)	2 (3.77)	NR
Gudibanda et al., 2024^42^	188	162 (86.17)	100 (53.19)	66 (35.12)	87 (46.28)	NR	19 (10.11)
Hassanein et al., 2025^43^	257	209 (81.32)	212 (82.49)	NR	77 (29.96)	NR	NR
Horii et al., 2024^45^	6,140	3,642 (59.32)	3,513 (57.221)	2,652 (43.19)	1,032 (16.90)	449 (7.31)	832 (13.55)
van Houtum et al., 2024^46^	187	152 (81.28)	8 (4.27)	NR	117 (62.57)	NR	NR
Ishiguro and Nishimura, 2025^47^	169	NR	NR	5	NR	NR	NR
Jain et al., 2023^48^	182	103 (56.59)	70 (38.46)	NR	49 (26.92)	1 (0.55)	NR
Janić et al., 2023^49^	20	20 (100)	10 (50)	NR	20 (100)	NR	NR
Rodríguez Jiménez et al., 2024^50^	33	25 (75.76)	15 (45.45)	24 (72.73)	6 (18.18)	NR	NR
Kick et al., 2024^52^	185	89 (48.11)	24 (12.97)	NR	13 (7.02)	1 (0.54)	NR
Lv et al., 2024^56^	5,485	4,020 (73.29)	1,777 (32.39)	NR	NR	NR	NR
Manti et al., 2025^57^	445	269 (60.44)	30 (6.74)	NR	10 (2.24)	NR	NR
Moreno-Pérez et al., 2024^58^	1,018	806 (79.17)	537 (52.75)	281 (27.60)	93 (9.13)	NR	NR
Oe et al., 2024^59^	30	13 (43.33)	14 (46.67)	3 (10)	8 (26.67)	1 (3.33)	3 (10)
Pinto et al., 2024^62^	23	16 (69.57)	9 (39.13)	7 (30.43)	6 (26.09)	2 (8.7)	NR
Ray et al., 2024^64^	80	74 (92.5)	66 (82.5)	23 (28.75)	14 (17.5)	NR	NR
Roy Chowdhury et al., 2024^65^	53	43 (81.13)	34 (64.15)	10 (18.88)	33 (62.26)	NR	NR
Sansone et al., 2025^22^	950	316 (33.26)	NR	278 (29.26)	634 (66.74)	NR	NR
Sanyal et al., 2025^67^	388	140 (36.08)	108 (27.83)	NR	91 (23.45	21 (5.41)	45 (11.5)
Saravanan et al., 2024^68^	333	266 (79.88)	147 (44.14)	NR	73 (21.92)	11 (3.30)	NR
Sato et al., 2024^69^	66	42 (63.64)	41 (62.12)	16 (24.24)	11 (16.67)	8 (12.12)	5 (7.58)
Trenas et al., 2025^71^	202	117 (57.92)	112 (55.44)	84 (41.58)	2 (0.99)	NR	NR
Weinreich et al., 2025^74^	96	73 (76.04)	72 (75)	19 (19.79)	NR	NR	NR
Williams et al., 2024^75^	76	19 (25)	19 (25)	5 (6.58)	13 (17.10)	2 (2.63)	NR
Yabe et al., 2025^77^	624	324 (51.92)	243 (38.94)	NR	137 (21.96)	49 (7.85)	44 (7.05)
Yamada et al., 2023^78^	88	39 (44.31)	60 (68.18)	23 (26.13)	10 (11.36)	5 (5.68)	13 (14.72)

*AGIs = alpha glucosidase inhibitors; NR = not reported; SGLT2i = sodium glucose transporter 2 inhibitor; SUs = sulphonylureas; TZDs = thiazolidinediones.*

*Forest plot for outcomes of change in (A) glycated haemoglobin (%) at 6 months of therapy with oral semaglutide; (B) proportion of participants achieving glycated haemoglobin <7%; (C) change in body weight (kg) at 6 months of therapy with oral semaglutide and (D) change in body mass index (kg/m²) at 6 months of therapy with oral semaglutide BMI = body mass index; CI = confidence interval; HbA1c = glycated haemoglobin; MRAW = mean using raw scale (raw mean difference); SD = standard deviation.*

### Weight and body mass index outcomes

**Figure 3:**
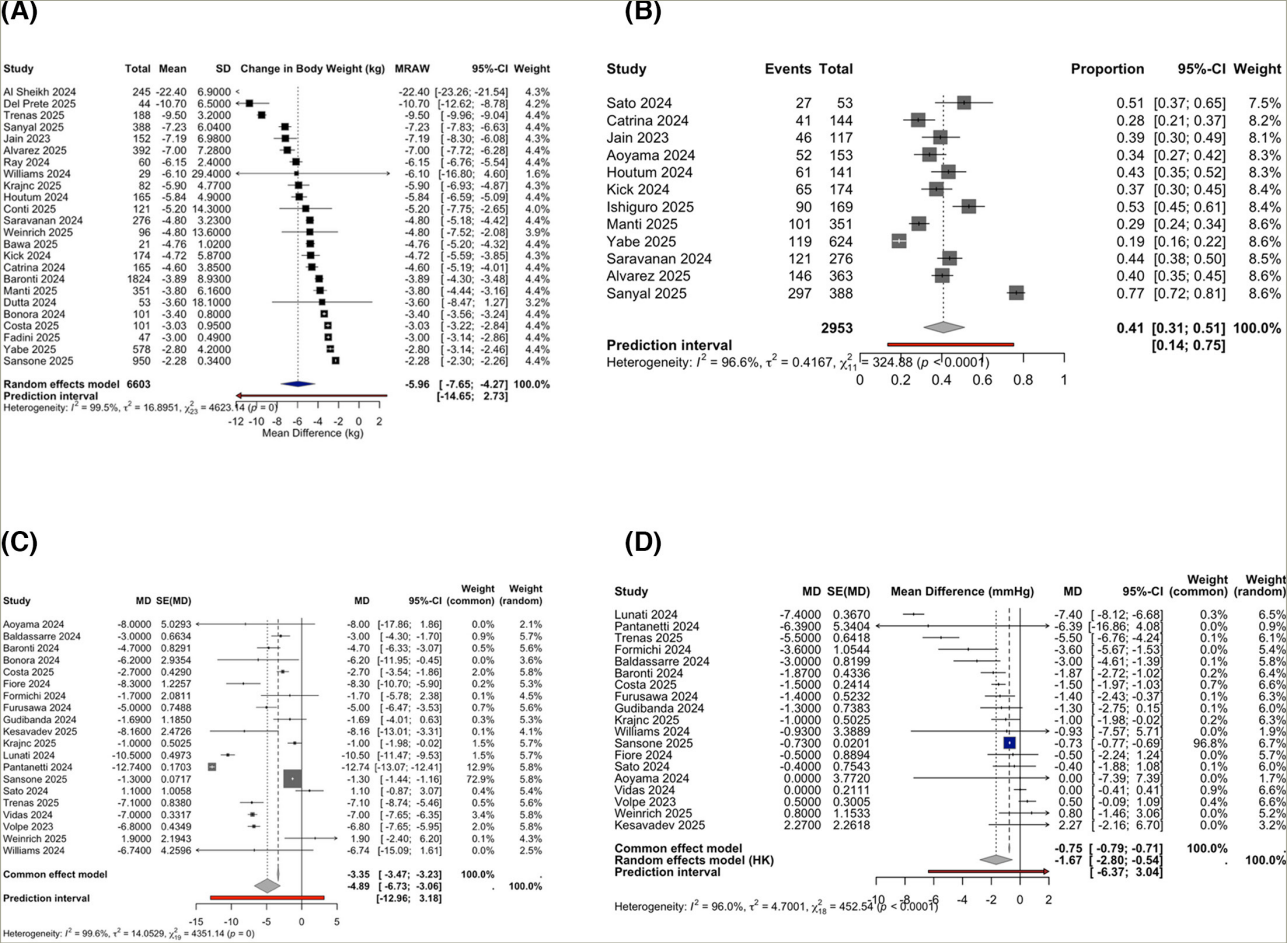
Impact of oral semaglutide on weight loss and blood pressure outcomes^22–74^

After 6 months’ use of oral semaglutide, pooled body weight decreased by -4.38 kg (95% CI: -5.56 to -3.20; I²=99.5%; *k*=28; *Figure 2C*), and BMI declined by -1.98 kg/m² (95% CI: -2.60 to -1.37; I²=94.9%; *k*=22; *Figure 2D*). After 12 months’ use of oral semaglutide, pooled body weight reduction was -5.96 kg (95% CI: -7.71 to -4.21; I²=99.5%; *k*=24; *Figure 3A*).^22–74^ The occurrence of ≥3% weight loss was 40.8% (95% CI: 31.2–51.2%; I²=96.6%; *k*=12; *Figure 3B*), and that of ≥5% weight loss was 30.6% (95% CI: 23.5–38.8%; I²=95.5%; *k*=22) following oral semaglutide use.

### Cardiometabolic parameters

SBP decreased by -4.89 mmHg (95% CI: -6.73 to -3.06; I²=99.6%; *k*=20; *Figure 3C*), and DBP decreased by -1.67 mmHg (95% CI: -2.80 to -0.54; I²=96.0%; *k*=19; *Figure 3D*) following oral semaglutide use. Total cholesterol decreased by an average of -15.04 mg/dL (95% CI: -19.96 to -10.12; I²=99.3%; *k*=17; SF-S7) following oral semaglutide use.

### Safety and tolerability

**Figure 4:**
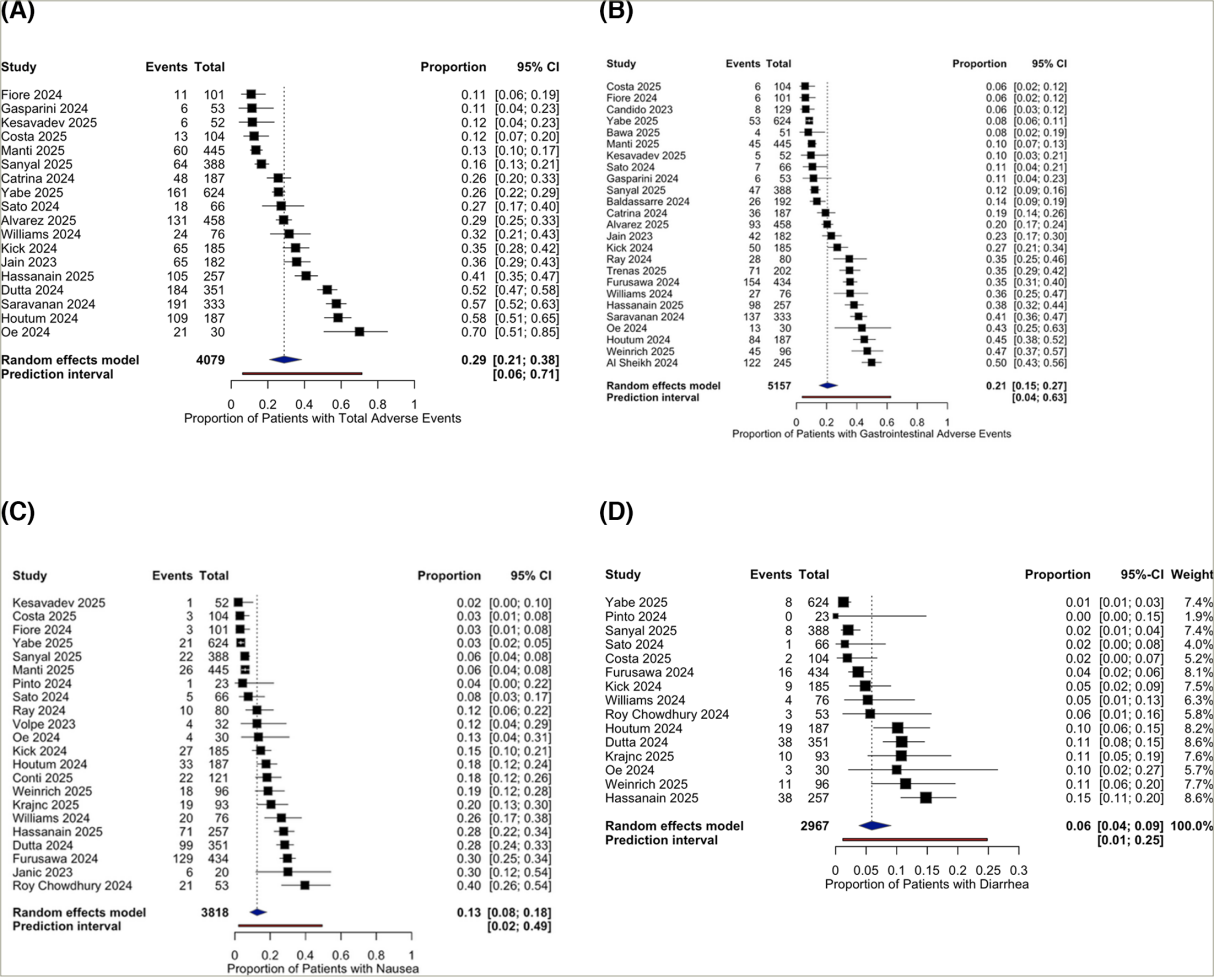
Adverse events outcomes with oral semaglutide^22–79^

Across real-world cohorts, TAEs occurred in 28.9% of patients (95% CI: 20.9–38.4%; I²=96.3%; *k*=18; *Figure 4A*).^22–79^ Gastro-intestinal (GI) adverse events were observed in 20.5% of patients (95% CI: 15.0–27.4%; I²=95.9%; *k*=25; *Figure 4B*), including nausea (12.5%; 95% CI: 8.5–18.1%; I²=94.4%; *k*=22; *Figure 4C*) and diarrhoea (5.9%; 95% CI: 3.8–9.1%; I²=84.3%; *k*=15; *Figure 4D*). Hypoglycaemia occurred in 2.2% of patients (95% CI: 1.1–4.3%; I²=69.3%; *k*=14; SF-S8), with severe hypoglycaemia in 0.9% of patients (95% CI: 0.4–1.9%; I²=66.1%; *k*=12; SF-S9). Discontinuation due to adverse drug reactions (ADRs) was observed in 8.7% of patients (95% CI: 6.0–12.3%; I²=75.6%; *k*=31; SF-S10), and dose reduction due to ADRs was observed in 4.2% of patients (95% CI: 2.0–8.5%; I²=84.3%; *k*=8; SF-S11).

### Subgroup analysis of Asian cohorts compared with the rest of the world

Data from 13 studies (1,714 patients) from Asia were compared with data from 21 studies (5,571 patients) from RoW with regard to HbA1c reduction at 6 months of oral semaglutide therapy.^14,16–20,23,25,28,30–35,41–43,47,50–53,55–57,61,62,64,67,70^ Asians had a higher HbA1c reduction of -1.25% (95% CI: -1.82 to -0.68; I^2^=99.4%) compared with -1.04% (95% CI: -1.32 to -0.76; I^2^=96.9%) in patients from RoW (SF-S12). However, the observation was not statistically significant (p=0.39).^14–21,23–70^

Data from 14 studies (1,772 patients) from Asia were compared with data from 15 studies (3,185 patients) from RoW with regard to body weight reduction at 6 months of oral semaglutide therapy.^14,16,18–20,23,25,27,28,30,32,34,35,41–43,47,51,53,54,56,57,61,64,65,67,70^ Asians had a marginally higher body weight reduction of -4.45 kg (95% CI: -6.58 to -2.33; I^2^=99.7%) compared with -4.27 kg (95% CI: -5.59 to -2.96; I^2^=91.8%) in patients from the RoW (Figure S13).^14,16,18–20,23,25,27,28,30,32,34,35,41–43,47,51,53,54,56,57,61,64,65,67,70^ However, the observation was not statistically significant (p=0.856).

*Forest plots for changes in (A) body weight (kg) at 12 months of therapy with oral semaglutide; (B) occurrence of ≥3% weight loss with oral semaglutide; (C) change in systolic blood pressure (mmHg) and (D) change in diastolic blood pressure (mmHg)*

*CI = confidence interval; HK = Hartung-Knapp method for adjustment of confidence intervals in random-effects meta-analysis; MD = mean difference; MRAW = mean using raw scale (raw mean difference); SD = standard deviation; SE = standard error.*

Data from eight studies (983 patients) from Asia were compared with data from 14 studies (3,088 patients) from RoW with regard to BMI reduction at 6 months of oral semaglutide therapy.^16,19,23,25,27,28,30,32,34,41–43,47,53,55–57,61,64,65,67^ Asians had a higher BMI reduction of -2.28 kg/m^2^ (95% CI: -3.91 to -0.65; I^2^=95.7%) compared with -1.82 kg (95% CI: -2.41 to -1.22; I^2^=93.5%) in patients from RoW (Figure S14). However, the observation was not statistically significant (p=0.571).^16,19,23,25,27,28,30,32,34,41–43,47,53,55–57,61,64,65,67^

Data from seven studies (1,624 patients) from Asia were compared with data from 17 studies (5,753 patients) from RoW with regard to HbA1c reduction at 12 months of oral semaglutide therapy.^14,15,19–21,24,26–29,38,40,44,49,56,59,60,63,66,67,69^ Asians had a higher HbA1c reduction of -1.58% (95% CI: -2.34 to -0.82; I^2^=99.7%) compared with -0.99% (95% CI: -1.21 to -0.78; I^2^=94.2%) in patients from the RoW (Figure S15). However, the observation was not statistically significant (p=0.154).^14,15,19–21,24,26–29,38,40,44,49,50,56,59,60,63,66,67,69^

Data from seven studies (1,621 patients) from Asia were compared with data from 17 studies (4,982 patients) from RoW with regard to body weight reduction at 12 months of oral semaglutide therapy.^14,15,19–21,24,26–29,38,40,44,49,50,56,59,60,63,66,67,69^ Asians had a higher body weight reduction of -7.45 kg (95% CI: -13.75 to -1.15; I^2^=99.7%) compared with -5.25 kg (95% CI: -6.45 to -4.06; I^2^=99.2%) in patients from RoW (Figure S16). However, the observation was not statistically significant (p=0.463).^14,15,19–21,24,26–29,38,40,44,45,49,56,59,60,63,66,67,69^

Data from eight studies (1,239 patients) from Asia were compared with data from 21 studies (6,566 patients) from RoW with regard to percentage of patients able to achieve the glycaemic target of HbA1c <7% on oral semaglutide therapy.^20,25,36,39,41–43,52,53,55,56,59,60,62,64–67,70^ The percentage of Asians able to achieve HbA1c <7% was 49.6% (95% CI: 30.1–69.3; I^2^=97.1%) compared with 56.6% (95% CI: 51.5–61.7; I^2^=91.6%) in patients from RoW (Figure S17).^20,25,36,39,41–43,52,53,55,56,59,60,62,64–67,70^

The comparison of changes in SBP, DBP and total cholesterol in Asians compared with RoW has been elaborated in SF-S18, SF-S19 and SF-S20, respectively. The occurrence of different adverse events was largely similar in Asians compared with RoW.^14–21,23–70^ The occurrence of total AEs, gastrointestinal AEs, nausea, diarrhoea, hypoglycaemia, severe hypoglycaemia, need for oral semaglutide dose reduction and the need for total stoppage of oral semaglutide has been elaborated in SF-S21, SF-S22, SF-S23, SF-S24, SF-S25, SF-S26, SF-S27 and SF-S28, respectively.

## Discussion

The global burden of T2D and obesity continues to escalate and contributes to the increasing prevalence of metabolic syndrome, cardiovascular disease and overall morbidity and mortality worldwide. The rise of these twin epidemics has led to an increased demand for agents that combine glycaemic lowering with significant weight loss, cardiovascular safety and patient convenience. Oral semaglutide, the first orally active GLP-1RA, is a potent and convenient agent with benefits for glycaemic control, as well as weight loss.

A literature review revealed that several meta-analyses of randomized controlled trials (RCTs) have been published on oral semaglutide.^13,81–85^ Their outcomes have been elaborated in *Table 3*.^13,81–85^ Since its availability for clinical use, many real-world studies (RWSs) have been published on the use of oral semaglutide.^22,79,80^ Two narrative reviews have been published to date evaluating the RWS on oral semaglutide without any quantitative analysis.^5,86^ No meta-analysis has been published to date quantitatively analysing the efficacy and safety of oral semaglutide use in the real-world setting. In addition, regional differences in treatment response and tolerability are unexplored; therefore, we also analysed subgroups based on studies from Asia and the rest of the world (RoW) to evaluate potential variations.

*Occurrence of different adverse events among oral semaglutide users, specifically (A) total adverse events, (B) gastrointestinal adverse events, (C) nausea and (D) diarrhoea CI = confidence interval.*

Real-world data reflect a more heterogeneous pool than those usually enrolled in RCTs. Our study represents the largest SRM of real-world data on oral semaglutide, including 59 studies and 28,479 adults with T2D.^13–21,23–70,84,85^

Our pooled analysis demonstrates that oral semaglutide use in real-world practice is consistently associated with significant improvement in glycaemic control and body weight. Additionally, it exerts favourable effects on blood pressure and lipid parameters. Despite differences in baseline control, comorbidities, adherence and background therapy, nearly half of the patients achieved optimal glycaemic control and weight reduction, which re-enforces the efficacy of semaglutide in real-world scenarios.

**Table 3:** Summary of outcomes of meta-analysis of data from randomized controlled trials on the use of oral semaglutide^13,81–85^

Authors	Objective	Number of RCTs	Number of participants	Key outcomes
Rebelo et al., 2025^81^	CV outcomes	5	OS=6,935; P=6,940	Reduced CV events; RR, 0.86 (95% CI: 0.78–0.95; p=0.0029; I^2^=0%)
Wang et al., 2025^82^	Asians versus non-Asians outcomes in T2D	10	7,817	Superior efficacy in HbA1c reduction; rapid action in Asians
Zhang et al., 2024^83^	Efficacy in T2D	10	9,541	Good efficacy and safety w.r.t placebo as well as active controls
Li et al., 2023^84^	Efficacy in T2D	11	9,821	HbA1c reduction with Sema7 and Sema14 was 1.06% (95% CI: 0.81–1.30) and 1.10% (95% CI: 0.88–1.31) w.r.t placebo
Alhindi and Avery, 2022^13^	Oral versus sc semaglutide, other GLP-1RAs; NMA	12	6,840	OS was non-inferior to sc sema and superior to placebo and other GLP-1RAs in reducing HbA1c and body weight
Avgerinos et al., 2020^85^	Oral sema versus placebo, liraglutide, empagliflozin and sitagliptin in T2D	11	9,890	Compared with placebo, OS reduced all-cause mortality (OR: 0.58; 95% CI: 0.37–0.92) and CV mortality (OR: 0.55; 95% CI: 0.31–0.98)

*CI = confidence interval; CV = cardiovascular; GLP-1RAs = glucagon-like peptide 1 receptor agonists; HbA1c = glycated haemoglobin; NMA = network meta-analysis;*

*OR = odds ratio; OS = oral semaglutide; P = placebo; RCTs = randomized controlled trials; RR = risk ratio; sc = subcutaneous; sema = semaglutide; T2D = type 2 diabetes; w.r.t = with regard to.*

In comparison, meta-analysis of RCTs on oral semaglutide, such as the PIONEER-1 (Efficacy and Safety of Oral Semaglutide Versus Placebo in Subjects With Type 2 Diabetes Mellitus Treated With Diet and Exercise Only; ClinicalTrials. gov identifier: NCT02906930), has reported HbA1c reductions of -1.4% and weight reduction of -2.6 kg with a 14 mg once-daily dose of oral semaglutide.^14^ Comparable outcomes have been reported in PIONEER-2 (empagliflozin comparator on a background of metformin therapy) and PIONEER-3 (sitagliptin comparator).^15,16^ The near similarity of effect sizes between RCTs and real-world evidence demonstrates the applicability of clinical trials to the real-world environment, even when patient adherence and follow-up are less stringent.^14–21,23–73^ The persistence of benefits at 12 months in our analysis further supports the long-term durability of oral semaglutide therapy, as observed in extension phases of RCTs (*Figure 3A*, Supplementary Material 2 *[S13]*). However, unlike PIONEER-5, which specifically included participants with moderate chronic kidney disease (CKD), our data set mainly comprised individuals without CKD, limiting applicability to this subgroup.^87^

The safety profile of oral semaglutide remained reassuring. Gastrointestinal adverse events were the most reported, but they were generally mild and transient. SAEs and severe hypoglycaemia were uncommon, aligning with the observations from landmark clinical trials.^14^ This real-world evidence has shown the effectiveness of oral semaglutide across wide spectra of patients, which are often underrepresented in RCTs, including elderly individuals, those with long-standing T2D and those on multiple drugs (*Figures 2 and 3*, Supplementary Material 2 *[S12-17]*). The magnitude of HbA1c and weight reduction suggests that early initiation of oral semaglutide can be an effective alternative for patients averse to injectable GLP-1RAs. Effects of semaglutide on blood pressure and total cholesterol highlight its holistic cardiometabolic benefits (*Figure 3*).

Subgroup analysis comparing Asian versus RoW revealed numerically greater glycaemic and weight improvement in Asian cohorts; however, these differences were not statistically significant. A meta-analysis by Kim et al. reported modest but statistically significant additional HbA1c reduction in Asian-predominant GLP-1RA trials.^88^ This has been explained by ethnic differences in β-cell function, BMI and background therapy.^88^ Our real-world subgroup data reinforce that semaglutide maintains comparable efficacy across geographical and ethnic boundaries.

The key strength of this study is that, to our knowledge, it is the largest global real-world meta-analysis on oral semaglutide to date, covering >27,000 patients across diverse real-world settings. The inclusion of heterogeneous populations provides a multifaceted view of how oral semaglutide performs in routine clinical settings. Another key strength is the use of robust random-effects modelling, which provides statistically relevant pooled estimates despite heterogeneity.

Limitations include the heterogeneity of the included studies, reflecting variations in dose, duration and follow-up. Most studies were observational, with risk of potential confounding by indication and incomplete reporting of adherence. Additionally, heterogeneity remained high for most outcomes, indicating differences in study design and patient population.

## Conclusion

This analysis shows that the real-world data on semaglutide are similar to the evidence demonstrated in major RCTs. Despite its limitations, the results of this meta-analysis reaffirm the utility and consistency of the effect of oral semaglutide across different patient groups. These findings bridge the gap between trial outcomes and real-world efficacy and strengthen the role of oral semaglutide as an early therapeutic option for diabetes management, especially in the obese subset.
